# Proteomic analysis of cerebrospinal fluid from children with central nervous system tumors identifies candidate proteins relating to tumor metastatic spread

**DOI:** 10.18632/oncotarget.17579

**Published:** 2017-05-03

**Authors:** Filippo Spreafico, Italia Bongarzone, Sara Pizzamiglio, Ruben Magni, Elena Taverna, Maida De Bortoli, Chiara M. Ciniselli, Elena Barzanò, Veronica Biassoni, Alessandra Luchini, Lance A. Liotta, Weidong Zhou, Michele Signore, Paolo Verderio, Maura Massimino

**Affiliations:** ^1^ Pediatric Oncology Unit, Department of Hematology and Pediatric Hematology-Oncology, Fondazione IRCCS Istituto Nazionale dei Tumori, Milan, Italy; ^2^ Proteomics Laboratory, Department of Experimental Oncology and Molecular Medicine, Fondazione IRCCS Istituto Nazionale dei Tumori, Milan, Italy; ^3^ Unit of Medical Statistics, Biometry and Bioinformatics, Department of Applied Research and Technological Development, Fondazione IRCCS Istituto Nazionale dei Tumori, Milan, Italy; ^4^ Center for Applied Proteomics and Molecular Medicine, George Mason University, Manassas, VA, USA; ^5^ Department of Hematology, Oncology and Molecular Medicine, Istituto Superiore di Sanità, Rome, Italy

**Keywords:** proteomic analysis, cerebrospinal fluid, pediatric central nervous system tumors, liquid chromatography/electrospray tandem mass spectrometry, protein-based biomarker

## Abstract

Central nervous system (CNS) tumors are the most common solid tumors in childhood. Since the sensitivity of combined cerebrospinal fluid (CSF) cytology and radiological neuroimaging in detecting meningeal metastases remains relatively low, we sought to characterize the CSF proteome of patients with CSF tumors to identify biomarkers predictive of metastatic spread. CSF samples from 27 children with brain tumors and 13 controls (extra-CNS non-Hodgkin lymphoma) were processed using core-shell hydrogel nanoparticles, and analyzed with reverse-phase liquid chromatography/electrospray tandem mass spectrometry (LC-MS/MS). Candidate proteins were identified with Fisher's exact test and/or a univariate logistic regression model. Reverse phase protein array (RPPA), Western blot (WB), and ELISA were used in the training set and in an independent set of CFS samples (60 cases, 14 controls) to validate our discovery findings. Among the 558 non-redundant proteins identified by LC-MS/MS, 147 were missing from the CSF database at http://www.biosino.org. Fourteen of the 26 final top-candidate proteins were chosen for validation with WB, RPPA and ELISA methods. Six proteins (type 1 collagen, insulin-like growth factor binding protein 4, procollagen C-endopeptidase enhancer 1, glial cell-line derived neurotrophic factor receptor α2, inter-alpha-trypsin inhibitor heavy chain 4, neural proliferation and differentiation control protein-1) revealed the ability to discriminate metastatic cases from controls. Combining a unique dataset of CSFs from pediatric CNS tumors with a novel enabling nanotechnology led us to identify CSF proteins potentially related to metastatic status.

## INTRODUCTION

Central nervous system (CNS) neoplasms are the most common solid tumors in childhood, and the first cause of tumor-related death in this age group. The histotypes most frequently diagnosed are low-grade gliomas and embryonic tumors, which respectively account for 50% and 20% of CNS tumors in children under 15 years of age. Several studies have shown that pediatric brain tumors often differ in their pathogenesis and biology from the adult counterpart, even if their histological features are indistinguishable [1]. Molecular classification into subgroups has proved an important issue for some tumors, such as medulloblastoma, and is under critical evaluation [2]. Though histologically heterogeneous, pediatric CNS tumors share mutual key diagnostic and treatment issues, that mainly concern how to improve the therapeutic index [3, 4].

Malignant cells in the cerebrospinal fluid (CSF) on cytology are predictive of a worse outcome in children with high-grade glioma and medulloblastoma [4–10], and indeed indicate late-stage disease. CSF cytology has a low sensitivity (<50%), however, despite its high specificity (>95%), and only approximately 10-20% of CSF specimens are positive for tumor cells [9].

The CSF is generated by blood filtration in the choroid plexus and by diffusion from the brain extracellular matrix into the ventricles, and it is considered a source of proteins equally important to tumor interstitial fluid [11]. Several CSF biomarkers have already been identified in various non-oncologic diseases and are already used in clinical practice [12]. For inoperable tumors, such disease biomarkers would be useful for orienting clinical decisions and assessing treatment response (together with MRI). For patients with surgically resectable tumors, they could shed light on prognosis and the risk of disease recurrence [13]. Other researchers have demonstrated in animal models that the CSF protein signature can preclinically detect the presence of a brain tumor, and suggested that the CSF proteome may be altered already at the earliest stages of tumorigenesis [14].

The identification of CSF biomarkers may be hampered by several physiological and technical issues, including low protein levels (0.3 to 0.7 mg/mL), broad protein concentration span (up to around 12 orders of magnitude), and the presence of highly abundant proteins masking the less abundant ones [15, 16].

The present paper describes a project designed to overcome these challenges and the results obtained, which support the identification of promising CSF biomarkers in pediatric CNS tumors.

## RESULTS

### Study populations

In Table 1 are reported the clinical characteristics of the 27 cases of cohort 1 (discovery cohort) and the 60 cases of cohort 2 (internal validation cohort).

**Table 1:** Clinical characteristics of cases in the two cohorts

Histological diagnosis	Cohort 1		Cohort 2	
	N	%	N	%
Atypical teratoid-rhabdoid tumor	1	3.7	4	6.7
Grade III ependymoma	4	14.8	1	1.7
Grade II ependymoma	1	3.7	5	8.3
Malignant glioma (anaplastic astrocytoma, glioblastoma, high-grade glioma NAS)	1	3.7	13	21.7
Anaplastic medulloblastoma	5	18.5	3	5.0
Classic/desmoplastic medulloblastoma	13	48.2	20	33.3
PNET	2	7.4	8	13.3
Anaplastic glioneuronal tumor			1	1.7
Germ-cell tumor			4	6.7
Choroid plexus carcinoma			1	1.7
**Timing of CSF sampling**	**N**	**%**	**N**	**%**
At diagnosis	21	77.8	45	75.0
During treatment	3	11.1	2	3.3
During follow-up	1	3.7	3	5.0
At progression/relapse	2	7.4	10	16.7
**CSF cytology**	**N**	**%**	**N**	**%**
Positive	3	11.1	5	8.3
Negative	24	88.9	54	90.0
Borderline			1	1.7
**Metastasis**	**N**	**%**	**N**	**%**
No	17	63.0	46	76.7
Yes	10	37.0	14	23.3
**Total**	**27**	**100.0**	**60**	**100.0**

### Protein identification by nanoparticle capture-MS analysis

**Figure 1:**
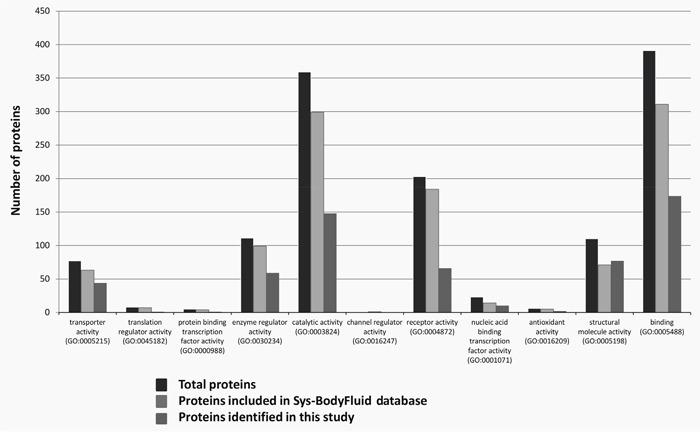
Overlap of the “CSF proteome” with the Sys-BodyFluid database Charts show the molecular function of the proteins identified in our CSF analysis and included in the Sys-BodyFluid Database identified using conventional analytical methods according to the Protein ANalysis THrough Evolutionary Relationships (PANTHER) classification system (version 8.1).

CSF samples from cohort 1 were pre-processed with poly(NIPAm/CB) core-poly(NIPAm-co-VSA) shell hydrogel nanoparticles (see Materials and Methods) and analyzed using liquid chromatography/electrospray tandem mass spectrometry (LC-MS/MS) to identify low-molecular-weight proteins. After label-free spectral counting (scaffold analysis, see Materials and Methods) of the LC-MS/MS results, a total of 558 National Center for Biotechnology Information (NCBI) annotated proteins were identified in both case and control groups (Supplementary Data 1, 2, and 3). This first list of proteins was searched against the public CSF proteome database (Sys-BodyFluid database [17]) to identify proteins not known to exist in the CSF. This database provides International Protein Index (IPI) identifiers corresponding to 1286 unique proteins (Supplementary Data 4). As our proteins were identified by means of NCBI accession numbers, the David gene accession conversion tool (https://david.ncifcrf.gov) was used to convert the NCBI gene identifier (GI) numbers into IPI identifiers to compare our list with the Sys-BodyFluid database (Supplementary Data 4). We were able to confidently (status: identical) convert 504 of the 558 NCBI GI accession numbers into 1926 IPI identifiers. We found 334 identifiers overlapping between the two lists, 1592 identifiers unique to our study, and 948 unique to the Sys-BodyFluid database (Figure 1 and Supplementary Data 4). Using the Panther system (http://www.pantherbd.org) to compare annotations corresponding to our IPI list with those corresponding to the Sys-BodyFluid database, we found 487 and 793 annotations, respectively, and 212 overlapping proteins (Supplementary Data 5), while 275 of the 487 annotations (56%) did not overlap.

### Functional classification of the proteins identified

The Panther system was used to conduct a functional classification analysis on 191 and 473 unique proteins found in the CSF samples of controls and cases, respectively (Supplementary Table 1). The results indicated 177 overlapping proteins. More than half of the CSF proteins were annotated as either extracellular or located in organelles (Supplementary Table 1). The major CSF proteins classes were signaling, hydrolase and receptor (Supplementary Table 2). Twenty-two molecular pathways were found in the CSF of controls, and 59 in the CSF of cases (Supplementary Figure 1, Supplementary Table 3), i.e. 37 molecular pathways were only seen in the CSF of cases.

### Selection of potentially relevant biomarkers

**Figure 2:**
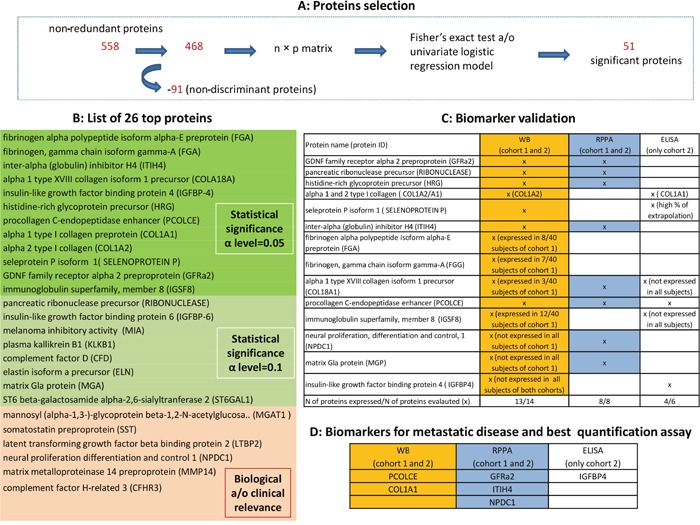
Workflow for the selection of CSF candidate biomarkers **(A)** show the proteins selection performed by the univariate analysis. **(B)** presents the 26 top-proteins selected based on both the biological and/or the statistical relevance: highlighted in green those statistically significant at alpha level of 0.05, in light green those statistically significant at alpha level of 0.10 and in orange those proteins selected considering biological and/or clinical relevance (MGAT1 was included because potentially involved in disease initiation and progression by reducing cell-cell adhesion and resulting in increased cell motility and migration [18]; somatostatin affects rates of neurotransmission in the central nervous system and proliferation of both normal and tumorigenic cells and it was included because it has anti-proliferative and pro-apoptotic effects [19]; latent transforming growth factor beta binding protein 2 (LTBP2) plays key roles in tumorigenesis through regulating TGFβ activity [20]; neural proliferation differentiation and control 1 plays a role in neuronal proliferation (NPDC1) [21]; matrix metalloproteinase 14 preproprotein (MMP14) is involved in tumor invasion (PMID: 20371345); complement factor H-related 3 (CFHR3) is prognostic in patients with neuroblastoma [22]. **(C)** reports the results of biomarker validation: proteins were measured using the indicated methods, both in cohort 1 (technical validation) and in an independent cohort 2 (internal validation). **(D)** reports the candidate biomarkers able to predict metastatic disease and recommended assay for their quantitative assessment.

Figure 2 shows the workflow used for the selection of biomarker candidates. Of the 558 non-redundant proteins indentified by the LC-MS/MS analysis, 486 were ultimately considered in the statistical analysis (highlighted in yellow in the Supplementary Data 3) because the other 91 were always expressed in all 40 subjects of cohort 1. Our statistical selection procedure led to the identification of a list of 51 proteins showing statistically significant results at alpha level of 0.05 in at least one of the comparisons performed (47/51 with a statistically significant result in the comparisons between cases and controls, and/or between metastatic cases and controls). Starting from this list, 12 proteins were considered in the subsequent analysis for their biological relevance, and another 8 proteins were selected by considering a significance threshold of 0.1, for a total of 20 proteins. Six more proteins were added to this panel in consideration of their biological and/or clinical relevance alone, for a total of 26 proteins, as listed in Table 2.

**Table 2:** List of 26 top proteins selected in the discovery phase

Clinical scenario considered	27 cases vs 13 controls		10 metastatic vs 13 controls		17 non-metastatic vs 13 controls		10 metastatic vs 17 non-metastatic	
	(486 proteins considered)		(335 proteins considered)		(389 proteins considered)		(437 proteins considered)	
protein	p-value logistic model	p-value Fisher	p-value logistic model	p-value Fisher	p-value logistic model	p-value Fisher	p-value logistic model	p-value Fisher
Fibrinogen alpha polypeptide isoform alpha-E preprotein	0.01	0.01	<0.001	<0.001	0.05	0.06	0.07	0.09
Pancreatic ribonuclease precursor	0.28	0.39	0.09	0.13	0.71	1	0.11	0.15
Fibrinogen. gamma chain isoform gamma-A	0.03	0.03	0.94	0.01	0.18	0.26	0.95	0.12
Inter-alpha (globulin) inhibitor H4	0.08	0.07	0.02	0.02	0.27	0.35	0.07	0.10
Alpha 1 type XVIII collagen isoform 1 precursor	0.1	0.12	0.95	0.05	0.38	0.44	0.96	0.26
Latent transforming growth factor beta binding protein 2	0.85	1	0.71	1	0.98	1	0.71	1.00
Insulin-like growth factor binding protein 4	0.96	0.04	0.95	0.01	0.96	0.24	0.09	0.10
Insulin-like growth factor binding protein 6	0.28	0.39	0.09	0.13	0.71	1	0.11	0.15
Histidine-rich glycoprotein precursor	0.96	0.04	0.95	0.01	0.96	0.24	0.09	0.10
Procollagen C-endopeptidase enhancer	0.96	0.02	0.96	0.07	0.95	0.02	0.78	1.00
Alpha 1 type I collagen preprotein	0.95	<0.001	0.94	<0.001	0.95	0.02	0.22	0.26
Melanoma inhibitory activity	0.15	0.23	0.09	0.13	0.27	0.35	0.37	0.42
Plasma kallikrein B1	0.96	0.28	0.96	0.07	0.98	1	0.12	0.13
Neural proliferation, differentiation and control. 1	0.81	1	0.41	0.62	0.77	1	0.25	0.33
Alpha 2 type I collagen	0.95	<0.001	0.94	<0.001	0.95	0.02	0.22	0.26
Complement factor D	0.96	0.15	0.96	0.07	0.97	0.49	0.25	0.33
Elastin isoform a precursor	0.96	0.15	0.96	0.07	0.97	0.49	0.25	0.33
Seleprotein P isoform 1	0.38	0.64	0.09	0.13	0.84	1	0.05	0.05
Matrix Gla protein	0.28	0.39	0.09	0.13	0.71	1	0.11	0.15
GDNF family receptor alpha 2 preproprotein	0.81	1	0.2	0.34	0.41	0.56	0.05	0.05
Immunoglobulin superfamily, member 8	0.33	0.37	0.71	1	0.96	0.07	0.96	0.04
Matrix metalloproteinase 14 preproprotein	0.97	0.54	0.97	0.18	0.98	1	0.29	0.54
Complement factor H-related 3	0.97	1	0.97	0.18	.	.	0.97	0.13
ST6 beta-galactosamide alpha-2,6-sialyltranferase 2	0.96	0.28	0.96	0.07	0.98	1	0.12	0.13
Mannosyl (alpha-1,3-)-glycoprotein beta-1,2-N-acetylglucosaminyltransferase	0.97	1	0.97	0.18	.	.	0.97	0.13
Somatostatin preproprotein	0.97	0.54	0.97	0.18	0.98	1	0.29	0.54

### Biomarker validation

To validate the results of the discovery procedure, the selected proteins were measured using alternative methods, both in cohort 1 (technical validation) and in an independent cohort 2 (internal validation). In particular, 14 and 8 of the 26 selected top proteins underwent validation by Western blot (WB) and reverse phase protein array (RPPA) methods, respectively, on the grounds of antibody availability and reliability. Three cases in cohort 1 were excluded from the RPPA analyses due to a shortage of material. In addition, 6/26 proteins -alpha 1 type XVIII collagen (COL18A1), type 1 collagen (COL1A1), insulin-like growth factor binding proteins 4 (IGFBP4), immunoglobulin superfamily member 8 (IGSF8), procollagen C-endopeptidase enhancer 1 (PCOLCE), selenoprotein P - were tested using the enzyme-linked immunosorbent assay (ELISA) method, but only in cohort 2 due to a shortage of material for cohort 1 (see C of Figure 2 for details of the proteins examined with each technique). Of the 14 proteins assessed with WB, one (IGFBP4) was never expressed in either cohort, two (matrix Gla protein, MGP; neural proliferation and differentiation control protein-1, NPDC1) were expressed only in cohort 2, and four (fibrinogen alpha chain, FGA; fibrinogen gamma chain, FGG; COL18A1; IGSF8) were expressed in ≤ 30% of the samples from cohort 1. The 8 proteins examined using RPPA were expressed in both cohorts of patients. Of the 6 proteins tested using ELISA in cohort 2, three (COL1A1, IGFBP4, PCOLCE) provided reliable results.

### Technical validation

When the results obtained with LC-MS/MS analysis were compared with those obtained with WB or RPPA on the same samples from cohort 1, 8 proteins showed a statistically significant concordant result, and were therefore considered ‘technically’ validated. In detail, histidine-rich glycoprotein (HRG) and COL1A1 were technically validated with WB, while COL18A1, glial cell-line derived neurotrophic factor receptor alpha 2 (GFRα2), inter-alpha-trypsin inhibitor heavy chain 4 (ITIH4), MGP, and ribonuclease A were technically validated with RPPA. PCOLCE was technically validated with both methods.

### Internal validation

**Figure 3:**
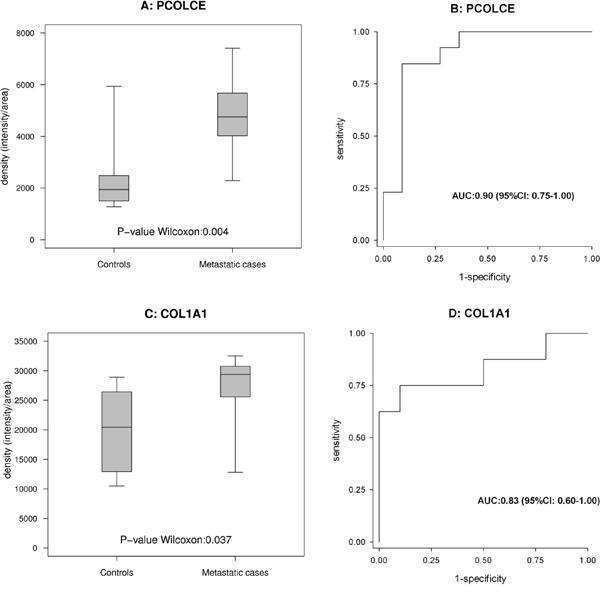
Proteins validated with Western blot analysis **(A** and **B)** show the box-plot for PCOLCE in 13 metastatic cases and 11 controls in cohort 2, and the corresponding ROC curves. **(C** and **D)** show the box-plot for COL1A1 in 8 metastatic cases and 10 controls in cohort 1 and the corresponding ROC curves.

**Figure 4:**
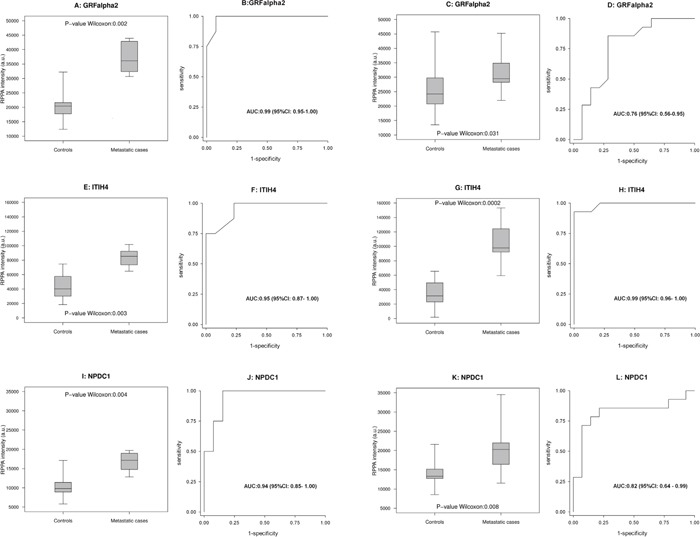
Proteins validated with RPPA analysis Box-plot **(A, E, I)** and ROC curve **(B, F, J)** for GFRalpha2, ITIH4 and NPDC1, respectively, in 8 metastatic cases and 13 controls in cohort 1. **(C, G, K, D, H and L)** likewise show the results for the same proteins in the 14 metastatic cases and 14 controls in cohort 2.

In this phase, the results obtained with WB or RPPA in cohort 1 were compared with those obtained using the same methods in cohort 2. Among the clinical scenarios considered to compare the protein expression profiles, the results were mainly validated for the purpose of comparing metastatic cases versus controls. Two proteins (PCOLCE, COL1A1) were validated with WB and three with RPPA (GFRα2, ITIH4, NPDC1), as shown in Figures 3 and 4, respectively. For PCOLCE, the statistically significant result observed in cohort 1 (Fisher's exact test p-value=0.013) was confirmed in cohort 2 (Wilcoxon's rank sum test p-value=0.004). For COL1A1, the statistically significant result observed in cohort 1 (Wilcoxon's rank sum test p-value=0.037) was confirmed in cohort 2 (Fisher's exact test p-value=0.002). The potential ability of the validated proteins to identify the presence of metastatic disease was examined by means of a ROC (Receiver Operating Characteristic) curve, and the corresponding AUC (area under the ROC curve) was found statistically significant (Figures 3 and 4).

**Figure 5:**
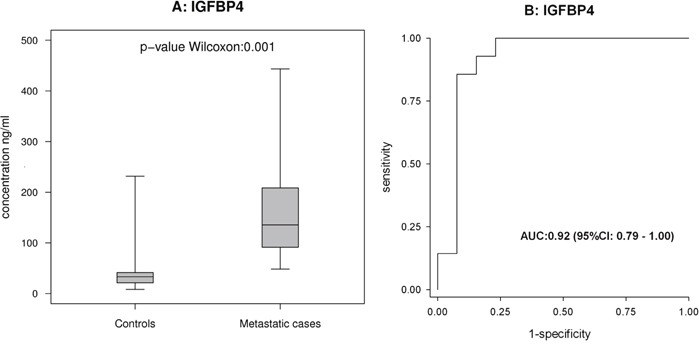
ELISA-based analysis of IGFBP4 in cohort 2 **(A and B)** show the box-plot for IGFBP4 in 14 metastatic cases and 13 controls in cohort 2, and the corresponding ROC curves.

Among the proteins examined using ELISA, IGFBP4 exhibited a statistically significant ability to discriminate between metastatic cases and controls (Figure 5).

For another four proteins a statistically significant concordant result was obtained for the same comparisons in cohorts 1 and 2, but using different methods. Specifically, for MGP the statistically significant result observed in cohort 1 with RPPA (Wilcoxon's rank sum test p-value=0.037) was confirmed in cohort 2 with WB (Fisher's exact test p-value=0.041). For selenoprotein P, FGA and FGG, the results of LC-MS/MS analysis were confirmed in cohort 2 using WB (selenoprotein P: Wilcoxon's rank sum test p-value=0.002; FGA: Wilcoxon's rank sum test p-value=0.034; FGG: Fisher's exact test p-value=0.046).

## DISCUSSION

The management of childhood CNS tumors relies mainly on histopathological analysis and neuroimaging, despite the complex genetic profile of these diseases. The intratumoral heterogeneity exhibited by these tumors demands more comprehensive methods of tumor characterization [2] and monitoring, and the ability to assess tumors over space and time. Circulating biomarkers hold promise as a non-invasive type of real-time liquid biopsy for providing dynamic information, and enabling adjustments to patient management to match the constantly evolving tumor.

We elected to study the CSF compartment (despite its known significant challenges) because it is considered the primary route for metastases from pediatric brain tumors [6, 9, 10], and because it contains numerous unique proteins. A specific aim of our analysis was to identify proteomic markers for predicting CSF metastatic dissemination, which is usually a late finding in the course of the disease using currently-available diagnostic procedures [9]. Alpha-fetoprotein and beta-human chorionic gonadotropin in germ-cell tumors provide a remarkable example of accurate CSF indicators capable of shedding light on diagnosis, risk profile and response to therapy [23].

We recognize that, although existing CSF biomarkers have proved useful in improving our understanding of a disease's pathogenesis, and expediting drug development for some neurological disorders (such as Alzheimer's and Parkinson's diseases [24]), the utility of CSF biomarkers in the field of neuro-oncology remains to be validated.

So far, isolated experiences have aimed for a comprehensive protein profiling of CSF specimens from children with brain tumors [13, 25–27]. Since pediatric glioma has key biological differences in tumorigenesis compared with the adult counterpart, biomarker information acquired in adult studies [28–30] is unlikely to be transferable directly to the pediatric population. Saratsis et al. analyzed CSF samples from 10 children with diffuse pontine glioma (using MS, linear trap quadrupole (LTQ)-Orbitrap), and identified selective upregulation of secreted cyclophillin A and dimethylarginase 1 [25]. De Bont et al. documented an overexpression of apolipoprotein A-II in the CSF from children with brain tumors using SELDI-TOF and ProteinChip arrays and WB [27].

CSF biomarker discovery poses some physiological and technical challenges, including: low protein levels (total protein concentrations from 1.5 to 6 mg/mL), dynamic range (up to approximately 12 orders of magnitude), and the presence of highly abundant proteins masking less abundant ones. Core-shell capturing hydrogel Nano traps can overcome these challenges, capturing, preserving, and concentrating candidate low-molecular-weight, low-abundance proteins in solution, in one step, and improving the effective sensitivity of MS by several orders of magnitude [31, 32].

After an initial non-targeted discovery phase, we were able to select 26 top proteins on the grounds of their statistical and biological relevance. Some of these proteins -but not all, depending on the availability of antibody pairs (and this might be seen as a limitation) underwent quantitative validation, and were scored in terms of their ability to distinguish CSF samples between metastatic cases and controls. We ultimately highlighted six proteins as potential biomarkers of meningeal spread. The results obtained were confirmed when only patients sampled in the diagnostic stage were considered in the statistical analysis (data not shown).

Coupling MS analysis with a core-shell nanoparticle capture technique probably enabled us to identify a significant number of low-weight and low-abundant proteins, not previously described in the CSF. The comparison between the list of proteins identified with our approach and the proteins previously identified in the CSF showed 147 proteins uniquely identified in this study. Our approach also found a proportion of extracellular proteins in the CSF higher than reportedly exist in the serum (14%), but similar to the proportion found in urine (38%) [23].

Some of the proteins selected (PCOLCE, COL1A1/2) are known to contribute to the formation of the extracellular matrix through fibroblast recruitment and collagen deposition [33, 34]. A concurrent increase in PCOLCE and COL1A1 may suggest a greater stromal production of the active type I collagen form [34]. Fibroblast and cerebral pericytes are required for angiogenesis, and some key genes expressed in fibroblast, including PCOLCE, are necessary for vessel formation [35, 36].

It is increasingly evident that it is not only the genetic aberrations in malignant cells that are crucial in the pathophysiology of cancer, but also the interactions between cancerous and nonmalignant cells, soluble factors, and other elements in the tumor microenvironment. Our data seem to support the hypothesis that the CSF proteome usefully reflects the brain tumor microenvironment.

It has recently been demonstrated that IGFBP4 (another protein selected in our study) has a role in promoting glioblastoma progression in adults, and in regulating factors relating to extracellular matrix formation and tumor invasion [37]. Pediatric oncologists have also recently pinpointed proteins related to the insulin-like growth factor network, on comparing proteomic profiles between medulloblastoma tissue collected at surgery and normal brain tissue [38]. ITIH4 acts as an acute-phase protein, and has been identified in proteomic studies as a serum biomarker that distinguishes women with breast cancer from healthy controls [39]. GFRα2 (from the GDNF family receptor α) has recently been described as a pituitary stem/progenitor marker, and found differentially expressed in pituitary tumors with a different biological behavior [40]. With these results in mind, the next step in our project will involve trying to correlate the selected proteins with different tumor histologies and anatomical sites of origin.

Studying the CSF proteome proved to be a difficult task. We were aware that it was imperative to follow our carefully-considered experimental design and data analysis strategies consistently in order to cope with all the various challenges. Some potential caveats need to be mentioned, however. The main limitation of our study lies in the small number of cases (and controls), though this is unavoidable and due to the relatively low prevalence of pediatric brain tumors by comparison with the adult counterpart. The present study nonetheless led to the creation of a unique bank of CSF samples from different pediatric CNS tumors, and analyzed 114 subjects in all.

Another caveat concerns how our control subjects were selected: it was important to have a robust control cohort, but our choosing to use CSF samples from children with extra-CNS non Hodgkin lymphoma may have introduced a bias due to the presence of lymphoma-related proteins [41]. On the other hand, it is very difficult to obtain CSF samples from healthy pediatric subjects for obvious ethical reasons. Furthermore, due to a shortage of CSF from the samples initially tested, we decided not to perform technical replicates in the MS analysis. We are confident in the robustness of our data despite the absence of technical replicates for two main reasons: our nanoparticle processing increases reproducibility of non-quantitative MS analysis; internal standards and native proteins known to exist in low concentration in CSF were consistently detected in the samples.

Much attention was paid during the sequential validation stages, but we were unable to validate our results in cohorts of samples from independent external sites, as originally planned. To our knowledge, only one study has demonstrated the feasibility (albeit with some difficulty) of coordinating multiple centers and centrally collecting and processing high-quality CSF samples for proteomic analysis [42]. The candidate biomarker emerging from said study, prostaglandin D2 synthase, was downregulated in the CSF from 33 children with medulloblastoma (compared with 25 controls), sampled at eight institutions across the USA.

In conclusion, processing CSF through nanoparticles enabled us to improve the detection of low-abundant and low-weight proteins, and we identified a pattern of CSF-secreted proteins that might discriminate patients with brain tumor from controls, and particularly those with metastatic tumor from controls. We also envisage proteomic technologies complementing an increasing array of brain imaging, immunohistochemical and cross-platform ‘omic’ data, for correlating many different facets of tumor growth.

## MATERIALS AND METHODS

### Sample collection and patient information

CSF samples were collected prospectively by lumbar puncture at the Pediatric Unit of our institution, with ethical approval (ref. 15/04/2009) and informed consent signed by the children's parents/guardians. The CSF samples were immediately frozen and kept at -80°C until analysis, under informed and standardized conditions. Samples were collected as part of standard staging procedures, both at initial diagnosis and at tumor relapse or progression. Patients’ demographics and medical records, histological diagnosis (centrally reviewed by the national reference pathologist), and CSF cytological findings were recorded for all patients (Table 1). The discovery cohort (cohort 1) included CSF samples from 27 children with CNS tumors (cases) and 13 control samples from children with extra-CNS non-Hodgkin lymphoma. The independent validation cohort (cohort 2) included CSF samples from 60 cases and 14 controls. In cohort 1 the median age of the patients was 85 months (range 8-429 months) for the cases, and 133 months for the controls (range 33-221 months); 63% and 77% of the cases and controls, respectively, were male. In cohort 2, the cases were a median 114 months old (range 18-583 months) and the controls 165 months (range 29-228 months); 58% and 64% of the cases and controls, respectively, were male.

### Synthesis and characterization of hydrogel NIPAm/AA CB-VSA core-shell particles

Hydrogel nanoparticles, poly(NIPAm-co-AA), were created by precipitation and polymerization, and covalently functionalized with Cibacron Blue F3GA (CB) dye. An outer shell containing vinylsulfonic acid (VSA) copolymer was created on the functionalized particles by means of a second polymerization reaction. The VSA shell increases the sieving capability of the NIPAm particles because it shields the core and its affinity bait groups from larger molecules that might compete with intended low-abundant, low molecular weight targets, for binding to the affinity bait in the core [27, 43].

### Sample preparation

CSF samples were centrifuged (7 minutes, 4°C, 16,100 rcf) and a 500 μL aliquot from each patient was diluted 1:2 with 50 mM Tris-HCl, pH 7.0. Two chemokines (IGFBP7 with a predicted molecular weight [MW] of 26 kDa; IGF-1 with a predicted MW of 7.6 kDa) were added to the CSF samples for standard internal process control purposes. CSF samples from cases were spiked with IGFBP7 and IGF-1 at a concentration of 2 ng/μL, 0.2 ng/μL, 0.04 ng/μL. Control samples were spiked with 0.2 ng/μL, 0.2 ng/μL, 0.4 ng/μL (Supplementary Figure 2). Diluted samples were subsequently incubated with 500 μL of nanoparticles for 30 minutes under slow rotation. After incubation, they were centrifuged (15 minutes, 25°C, 16,100 rcf), the supernatant was discarded and the particles were washed twice with 1 mL of MilliQ H_2_O, then centrifuged (15 minutes, 25°C, 16,100 rcf). The resulting pellet was resuspended with 600 μL of elution buffer (70% acetonitrile and 10% ammonium hydroxide) and incubated at room temperature for 20 minutes under slow rotation, then centrifuged (15 minutes, 25°C, 16,100 rcf). The elution step was repeated twice and the eluates pooled together and dried under a nitrogen flow. Samples were reduced by incubating them with a 10 mM dithiothreitol in 8 M urea for 30 minutes, and alkylated with 50 mM iodacetamide at room temperature in the dark for 20 minutes. To ensure the reproducibility of the digestion, trypsin was consistently used at a protease to total protein mass ratio of 1/10. Five μg of total protein were analyzed for each sample. The enzymatic digestion ran overnight with 0.5 μg sequencing grade trypsin (Promega) in 50 mM ammonium bicarbonate pH 8 at 37°C. Digestion was then stopped by adding 5μl of glacial acetic acid. Digested samples were then desalted with C-18 Zip Tips (Millipore). Final eluates from Zip Tips were then dried with a nitrogen evaporator. Samples were reconstituted in 6 μl of 0.1% formic acid added with 100 fmol of angiotensin 1.

### Mass spectrometry analysis

The digests were analyzed by reverse-phase LC-MS/MS using a LTQ-Orbitrap MS (Thermo Fisher Scientific, Waltham, MA) equipped with an in-line autosampler. Samples were loaded onto a reverse-phase C18 column (0.2 mm × 50 mm, Michrom BioResources, Auburn, CA). After sample injection, the column was washed for 5 minutes with mobile phase A (0.1% formic acid), and peptides were eluted using a linear gradient of 0% mobile phase B (0.1% formic acid, 80% acetonitrile) to 45% mobile phase B in 120 minutes. The MS was operated in a data-dependent mode in which each full MS scan was followed by five MS/MS scans in which the five most abundant molecular ions were dynamically selected and fragmented by collision-induced dissociation using a normalized collision energy of 30%. To reduce carryover, a blank was analyzed after every sample using a 30-minute double high-performance liquid chromatography (HPLC) gradient program. To test HPLC reproducibility, two standard samples (yeast enolase digest, Michrom BioResources) were analyzed using a 30-minute HPLC program after analyzing 15 CSF samples.

### Protein identification and biological data mining

MS data was collected from one single analysis per sample. Proteins were identified with SEQUEST. A stringent filter after SEQUEST analysis was applied. An estimated false discovery rate (FDR) of 1% was calculated, based on forward-reverse decoy. The data were searched against a fully tryptic indexed human protein database maintained by the NCBI with variable oxidized methionine and static carboxyamidomethylated cysteine modification. The search results were filtered using the following criteria: minimum XCorr=2.2 (+2), 3.5 (+3), minimum dCn=0.1, and a maximum precursor ion mass deviation of 15 ppm. An MS1-based comparative data analysis was run using BioSieve (Thermo and Vast Scientific). Spectral counting (MS2-based) analysis was done using Scaffold (Proteome Software Inc., Portland, OR). The gene ontology annotations for selected proteins were obtained using the Panther classification system 9.0 (http://www.pantherbd.org) [44]. The non-redundant CSF proteome was compared with the Sys-BodyFluid proteome database at http://lifecenter.sgst.cn/bodyfluid/ [17]. The Database for Annotation, Visualization and Integrated Discovery (David) (https://david.ncifcrf.gov) bioinformatic resource was used to convert protein identifiers [45].

### Western blot analysis

For each CSF sample, protein concentrations were measured with the bicinchoninic acid (BCA) assay (Bio-Rad Laboratories Srl, MI, IT). The protein content of the samples ranged from 0.25 μg/μl to 1.25 μg/μl, and 20 μg of each sample were loaded on 4-12% NuPAGE Bis-Tris gradient mini gels (Life Technologies, Grand Island, NY).

Protein electroblotting was performed following standard procedures, using antibodies for IGSF8, ITIH4, PCOLCE, FGA, FGG, GFRalpha2 and COL18A1 from Sigma-Aldrich Ltd (The Old Brickyard, New Road, Gillingham, Dorset SP8 4XT); for COL1A2, HRG, IGFBP4, MGP, NPDC1 and ribonuclease A from Abcam 330 (Cambridge Science Park, Cambridge CB4 0FL, UK); for selenoprotein P from Santa Cruz (10410 Finnell Street Dallas, Texas 75220 USA). WB bands were quantified with Quantity One software (Bio-Rad, USA) in terms of density (total intensity/area of volume).

### Reverse phase protein arrays

RPPA were obtained as described previously [46]. Samples were lysed using Tissue Protein Extraction Reagent (Thermo Scientific, Waltham, MA, USA) and diluted to 0.5 mg/mL with Novex Tris-Glycine SDS Sample Buffer 2X (Invitrogen Corporation, Carlsbad, CA, USA Thermo Scientific). Each lysate was spotted in 4-point two-fold dilution curves onto nitrocellulose-coated microscope slides (Grace Biolabs, Bend, OR, USA) using an Aushon Arrayer 2470 (Aushon Biosystems, Billerica, MA, USA). Slides were incubated with a single pre-validated primary antibody using DAKO Autostainer Plus (DAKO Corporation, Glostrup, Denmark). They underwent signal amplification (CSA kit, DAKO) and staining with streptavidin-conjugated IRDye680LT® (LI-COR Biosciences, Lincoln, NE, USA), before scanning on a Tecan Power Scanner (Männedorf, Switzerland) equipped with a customized emission filter to improve the IRDye680LT® fluorescence collection efficiency. Image analysis for spot recognition, quantification and normalization was carried out using MicroVigene 5.2 software (Vigene Tech Inc., Carlisle, MA, USA). For RPPA analysis, antibodies for COL18A1, GRFα2, ITIH4, PCOLCE were from Sigma-Aldrich Ltd (The Old Brickyard, New Road, Gillingham, Dorset SP8 4XT) and antibodies for HRG, MGP, NPDC1 and ribonuclease A were from Abcam 330 (Cambridge Science Park, Cambridge CB4 0FL, UK).

### Protein level testing by ELISA

The levels of the selected proteins in the CSF were measured using commercially-available immunoassay kits according to the manufacturer's instructions (MyBioSource – PO Box 153308, San Diego, CA, USA).

### Statistical analysis

We applied a first selection procedure to the list of unique proteins identified by the LC-MS/MS analysis to obtain a subset of proteins potentially predictive of disease. At this stage, each protein was dichotomized as present (1) or absent (0), and selected using filter methods based on Fisher's exact test and/or univariate logistic regression model [47], considering the cases and controls in cohort 1. The role of individual proteins was also investigated by considering another three different clinical scenarios involving: metastatic cases versus controls; localized tumor cases versus controls; and metastatic versus localized tumor cases. Finally, starting from the results obtained for each of the scenarios investigated, a list of top proteins judged to be statistically significant and/or with a relevant biological function was drawn up. Some proteins on this list were then assessed in the same cohort (cohort 1) using WB analysis and RPPA, and in the independent validation cohort (cohort 2) using WB, RPPA and ELISA, depending on the reliability and availability of the antibodies. The expression levels of each of these proteins obtained with the WB, RPPA and ELISA methods were then compared for the previously-considered clinical scenarios using Wilcoxon's rank sum test. Then, the predictive capability of each protein was assessed in terms of the AUC [48]. When there was a high proportion of samples with missing values (≥50%) for the expression level of a given protein, the comparison was drawn with Fisher's exact test considering the protein on a dichotomized scale (present/absent). Statistical analyses were run with the SAS software v. 9.2 (SAS Institute Inc., Cary, NC, USA).

## SUPPLEMENTARY DATAS, TABLES AND FIGURES















## Abbreviations

CNS: central nervous system
CSF: cerebrospinal fluid
LC-MS/MS: liquid chromatography/electrospray tandem mass spectrometry
MS: mass spectrometry
RPPA: reverse phase protein array
WB: Western blot
ELISA: enzyme-linked immunosorbent assay
ROC: receiver operating characteristic curve
AUC: area under the ROC curve
PNET: primitive neuroectodermal tumor
MRI: magnetic resonance imaging
NCBI: National Center for Biotechnology Information
HPLC: high performance liquid chromatography
VSA: vinylsulfonic acid
COL1A1: Type 1 collagen
COL18A1: Alpha 1 type XVIII collagen
IGFBP4: insulin-like growth factor binding protein 4
PCOLCE: procollagen C-endopeptidase enhancer 1
GFRα2: glial cell-line derived neurotrophic factor receptor α2
ITIH4: inter-alpha-trypsin inhibitor heavy chain 4
NPDC1: neural proliferation and differentiation control protein-1
IGSF8: immunoglobulin superfamily member 8
MGP: matrix Gla protein
FGA: fibrinogen alpha chain
FGG: fibrinogen gamma chain
HRG: histidine-rich glycoprotein
MW: molecular weight
