# Supplementary material for: Proteomic analysis of cerebrospinal fluid from children with central nervous system tumors identifies candidate proteins relating to tumor metastatic spread
Source: Oncotarget. 2017 May 3;8(28):46177–90. doi: 10.18632/oncotarget.17579 (PMC5542258; doi:10.18632/oncotarget.17579)
Supplement: Supplementary file 1 [file oncotarget-08-46177-s001.pdf]

Proteomic analysis of cerebrospinal fluid from children with central nervous system tumors identifies candidate proteins relating to tumor metastatic spread

SUPPLEMENTARY MATERIALS

Supplementary Data 1: List of proteins and peptides identified by mass spectrometry after the SEQUEST searcher for each sample

See Supplementary Data 1

Supplementary Data 2: Spectral count of the 558 identified proteins

See Supplementary Data 2

Supplementary Data 3: List of the 558 NCBI annotated proteins identified in both case and control groups after label free spectral counting (scaffold analysis) of the LC-MS/MS results

See Supplementary Data 3

Supplementary Data 4: Protein identifiers in column A were downloaded from CSF proteome database www.biosino.org/bodyfluid. This database provides International Protein Index (IPI) identifiers corresponding to 1286 unique proteins. As our proteins were identified by NCBI GI accession numbers, we used David gene ID conversion tool (https://david.ncifcrf.gov) to convert the NCBI GI accession numbers to IPI identifiers in order to compare our and Biosino lists (column B). We were able to confidently (status: identical) convert 504 of the 558 NCBI GI accession numbers to 1926 IPI identifiers. In column C the total list of IPI identifiers (3212, both databases); in column D 2878 unique identifiers. Thus 334 identifiers overlapped between the two lists, 1592 identifiers were unique (column E) for our study and 948 unique in Biosino database

See Supplementary Data 4

Supplementary Data 5: After the conversion from IPI identifiers to UniProt accession numbers we compared through PANTHER system Uniprot annotations included in Biosino database (column A) with those identified by us (Column B): 793 and 487 annotations, respectively (total annotations in column C, unique in column D). 212 uniprot accession number overlapped; 275 out of 487 annotations (56%) did not overlap Biosino database

See Supplementary Data 5

Supplementary Table 1: Cellular components according to the PANTHER database

See Supplementary File 1

Supplementary Table 2: Protein classes according to the PANTHER database

See Supplementary File 1

Supplementary Table 3: Molecular pathways according to the PANTHER database

See Supplementary File 1

Molecular pathways	Protein N°
Alzheimer disease-amyloid secretase pathway (P00003)	2
Alzheimer disease-presenilin pathway (P00004)	4
Alzheimer disease-presenilin pathway (P00004)	1
Angiogenesis (P00005)	2
Angiotensin II-stimulated signaling through G proteins and beta-arrestin (P05911)	2
Angiotensin II-stimulated signaling through G proteins and beta-arrestin (P05911)	1
Apoptosis signaling pathway (P00006)	1
Axon guidance mediated by netrin (P00009)	2
Axon guidance mediated by semaphorins (P00007)	1
Axon guidance mediated by Slit/Robo (P00008)	2
B cell activation (P00010)	2
Blood coagulation (P00011)	19
Blood coagulation (P00011)	14
BMP_signaling_pathway-drosophila (P06211)	1
Cadherin signaling pathway (P00012)	6
Cadherin signaling pathway (P00012)	3
CCKR signaling map (P06959)	4
CCKR signaling map (P06959)	2
Cytoskeletal regulation by Rho GTPase (P00016)	9
Cytoskeletal regulation by Rho GTPase (P00016)	1
De novo purine biosynthesis (P02738)	1
Dopamine receptor mediated signaling pathway (P05912)	1
DPP_signaling_pathway (P06213)	1
DPP-SCW_signaling_pathway (P06212)	1
EGF receptor signaling pathway (P00018)	4
Endothelin signaling pathway (P00019)	1
Enkephalin release (P05913)	1
Enkephalin release (P05913)	1
FAS signaling pathway (P00020)	1
FAS signaling pathway (P00020)	1
FGF signaling pathway (P00021)	3
General transcription by RNA polymerase I (P00022)	1
General transcription regulation (P00023)	1
Glycolysis (P00024)	3
Glycolysis (P00024)	1
Gonadotropin releasing hormone receptor pathway (P06664)	8
Gonadotropin releasing hormone receptor pathway (P06664)	2
Huntington disease (P00029)	8
Huntington disease (P00029)	3
Inflammation mediated by chemokine and cytokine signaling pathway (P00031)	13
Inflammation mediated by chemokine and cytokine signaling pathway (P00031)	3
Insulin/IGF pathway-mitogen activated protein kinase kinase/MAP kinase cascade (P00032)	1
Integrin signalling pathway (P00034)	15
Integrin signalling pathway (P00034)	3
Interferon-gamma signaling pathway (P00035)	1
Interleukin signaling pathway (P00036)	1
Ionotropic glutamate receptor pathway (P00037)	1
Metabotropic glutamate receptor group I pathway (P00041)	1
Metabotropic glutamate receptor group II pathway (P00040)	1
Metabotropic glutamate receptor group II pathway (P00040)	1
Metabotropic glutamate receptor group III pathway (P00039)	1
Methylmalonyl pathway (P02755)	1
Muscarinic acetylcholine receptor 1 and 3 signaling pathway (P00042)	1
Nicotine pharmacodynamics pathway (P06587)	1
Nicotinic acetylcholine receptor signaling pathway (P00044)	3
Nicotinic acetylcholine receptor signaling pathway (P00044)	1
Opioid prodynorphin pathway (P05916)	1
Opioid prodynorphin pathway (P05916)	1
Opioid proenkephalin pathway (P05915)	1
Opioid proenkephalin pathway (P05915)	1
p38 MAPK pathway (P05918)	1
p53 pathway (P00059)	2
Parkinson disease (P00049)	3
PDGF signaling pathway (P00047)	2
Plasminogen activating cascade (P00050)	7
Plasminogen activating cascade (P00050)	4
Pyruvate metabolism (P02772)	1
Ras Pathway (P04393)	2
SCW_signaling_pathway (P06216)	1
T cell activation (P00053)	2
TGF-beta signaling pathway (P00052)	2
Toll receptor signaling pathway (P00054)	2
Toll receptor signaling pathway (P00054)	1
Transcription regulation by bZIP transcription factor (P00055)	1
Vasopressin synthesis (P04395)	2
Vasopressin synthesis (P04395)	1
VEGF signaling pathway (P00056)	2
Vitamin D metabolism and pathway (P04396)	1
Vitamin D metabolism and pathway (P04396)	1
Wnt signaling pathway (P00057)	8
Wnt signaling pathway (P00057)	3

Supplementary Figure 1: Overview of the distribution of molecular functions in control (red) and cases (black) and shared (highlighted in orange) according to the PANTHER database.

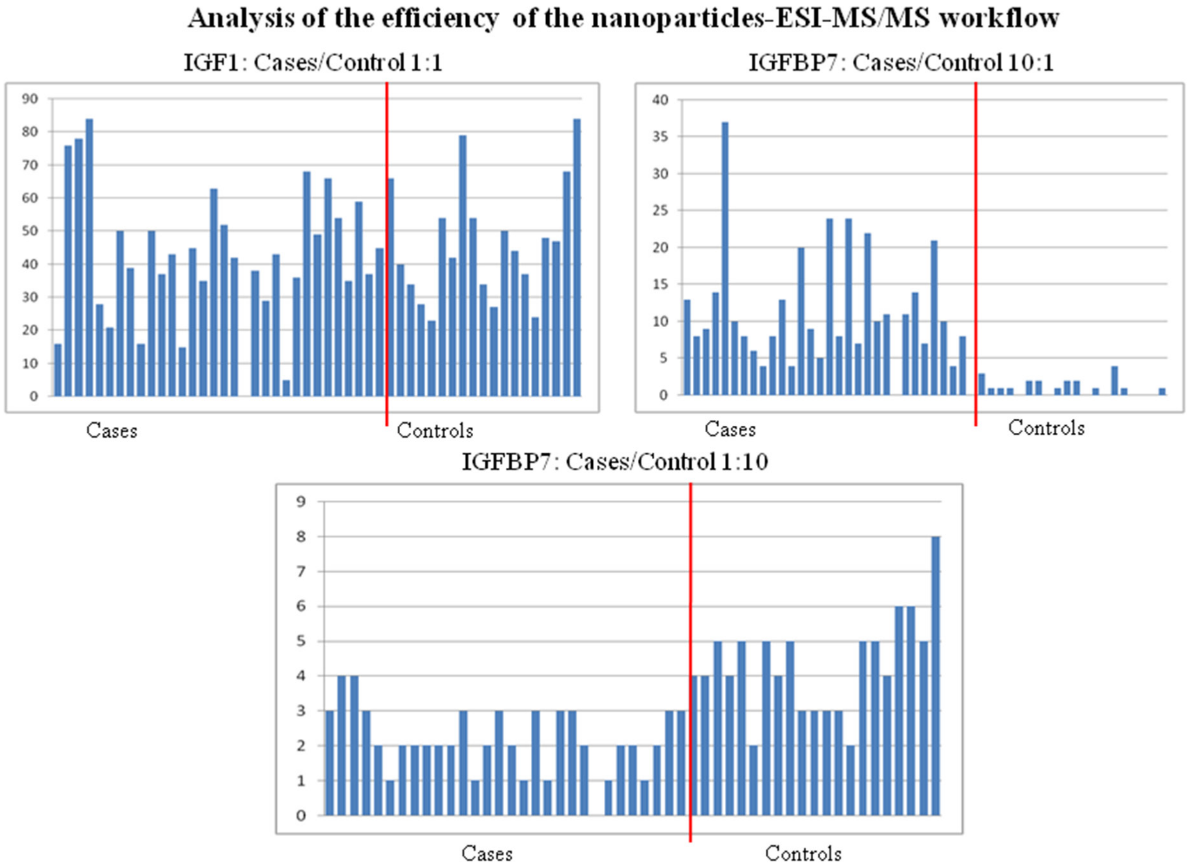

Supplementary Figure 2: Two chemokines IGFBP7 and IGF-1 were spiked in the CSF samples before nanoparticles incubation as control standards in order to evaluate the performance of nanoparticles harvesting. These chemokines were spiked in cases versus controls in a ratio of 10:1, 1:1, respectively. After Scaffold analysis, they were found to be differentially abundant with a ratio similar to the expected one, (actual ratio of 11.1 and 0.92) in brain tumor samples versus controls.
