# Supplementary material for: Proteomic analysis of cerebrospinal fluid from children with central nervous system tumors identifies candidate proteins relating to tumor metastatic spread
Source: Oncotarget. 2017 May 3;8(28):46177–90. doi: 10.18632/oncotarget.17579 (PMC5542258; doi:10.18632/oncotarget.17579)
Supplement: Supplementary file 7 [file oncotarget-08-46177-s007.docx]

**Additional supporting table.**

| **Supplementary Table 1:** **Cellular components according to the PANTHER database** | | | |
| --- | --- | --- | --- |
|  |  |  |  |
|  | **473 Proteins from CNS tumor patients** | **N** | **%** |
| 1 | Cell junction (GO:0030054) | 1 | 0.4 |
| 2 | Membrane (GO:0016020) | 14 | 5.9 |
| 3 | Macromolecular complex (GO:0032991) | 13 | 5.5 |
| 4 | Extracellular matrix (GO:0031012) | 27 | 11.4 |
| 5 | Cell part (GO:0044464) | 61 | 25.7 |
| 6 | Organelle (GO:0043226) | 44 | 18.6 |
| 7 | Extracellular region (GO:0005576) | 77 | 32.5 |
|  |  |  |  |
|  | **191 Proteins from controls** | **N** | **%** |
| 1 | Cell junction (GO:0030054) | 1 | 1.3 |
| 2 | Cembrane (GO:0016020) | 3 | 3.8 |
| 3 | Macromolecular complex (GO:0032991) | 5 | 6.4 |
| 4 | Extracellular matrix (GO:0031012) | 10 | 12.8 |
| 5 | Cell part (GO:0044464) | 14 | 17.9 |
| 6 | Organelle (GO:0043226) | 8 | 10.3 |
| 7 | Extracellular region (GO:0005576) | 37 | 47.4 |

| **Supplementary Table 2:** **Protein classes according to the PANTHER database** | | | | | | | | |
| --- | --- | --- | --- | --- | --- | --- | --- | --- |
|  |  |  |  |  |  |  |  |  |
|  | **CNS tumor patients** | **N** | **%** |  |  | **Controls** | **N** | **%** |
| 1 | Extracellular matrix protein (PC00102) | 26 | 4.4 |  | 1 | extracellular matrix protein (PC00102) | 11 | 4.2 |
| 2 | Protease (PC00190) | 42 | 7.1 |  | 2 | protease (PC00190) | 20 | 7.6 |
| 3 | Cytoskeletal protein (PC00085) | 46 | 7.8 |  | 3 | cytoskeletal protein (PC00085) | 9 | 3.4 |
| 4 | Transporter (PC00227) | 23 | 3.9 |  | 4 | transporter (PC00227) | 10 | 3.8 |
| 5 | Transmembrane receptor regulatory/adaptor protein (PC00226) | 1 | 0.2 |  | 5 | transferase (PC00220) | 2 | 0.8 |
| 6 | Transferase (PC00220) | 6 | 1.0 |  | 6 | oxidoreductase (PC00176) | 5 | 1.9 |
| 7 | Oxidoreductase (PC00176) | 12 | 2.0 |  | 7 | cell adhesion molecule (PC00069) | 17 | 6.5 |
| 8 | Lyase (PC00144) | 2 | 0.3 |  | 8 | ligase (PC00142) | 1 | 0.4 |
| 9 | Cell adhesion molecule (PC00069) | 36 | 6.1 |  | 9 | nucleic acid binding (PC00171) | 4 | 1.5 |
| 10 | Ligase (PC00142) | 3 | 0.5 |  | 10 | signaling molecule (PC00207) | 28 | 10.6 |
| 11 | Nucleic acid binding (PC00171) | 10 | 1.7 |  | 11 | enzyme modulator (PC00095) | 26 | 9.9 |
| 12 | Signaling molecule (PC00207) | 63 | 10.7 |  | 12 | calcium-binding protein (PC00060) | 11 | 4.2 |
| 13 | Enzyme modulator (PC00095) | 56 | 9.5 |  | 13 | defense/immunity protein (PC00090) | 19 | 7.2 |
| 14 | Calcium-binding protein (PC00060) | 25 | 4.2 |  | 14 | hydrolase (PC00121) | 32 | 12.2 |
| 15 | Defense/immunity protein (PC00090) | 34 | 5.8 |  | 15 | transfer/carrier protein (PC00219) | 21 | 8.0 |
| 16 | Hydrolase (PC00121) | 61 | 10.3 |  | 16 | membrane traffic protein (PC00150) | 1 | 0.4 |
| 17 | Transfer/carrier protein (PC00219) | 33 | 5.6 |  | 17 | phosphatase (PC00181) | 5 | 1.9 |
| 18 | Membrane traffic protein (PC00150) | 3 | 0.5 |  | 18 | transcription factor (PC00218) | 1 | 0.4 |
| 19 | Phosphatase (PC00181) | 8 | 1.4 |  | 19 | cell junction protein (PC00070) | 1 | 0.4 |
| 20 | Transcription factor (PC00218) | 4 | 0.7 |  | 20 | surfactant (PC00212) | 2 | 0.8 |
| 21 | Cell junction protein (PC00070) | 6 | 1.0 |  | 21 | structural protein (PC00211) | 7 | 2.7 |
| 22 | Surfactant (PC00212) | 5 | 0.8 |  | 22 | isomerase (PC00135) | 2 | 0.8 |
| 23 | Structural protein (PC00211) | 23 | 3.9 |  | 23 | receptor (PC00197) | 28 | 10.6 |
| 24 | Kinase (PC00137) | 3 | 0.5 |  |  |  |  |  |
| 25 | Storage protein (PC00210) | 1 | 0.2 |  |  |  |  |  |
| 26 | Receptor (PC00197) | 57 | 9.7 |  |  |  |  |  |
| 27 | Isomerase (PC00135) | 1 | 0.2 |  |  |  |  |  |

| **Supplementary Table 3**: **Molecular pathways according to the PANTHER database** | | | |  |  |  |  |
| --- | --- | --- | --- | --- | --- | --- | --- |
|  | | | |  |  |  |  |
|  | **CNS tumor patients** | **N** | **%** |  | **Controls** | **N** | **%** |
| 1 | DPP_signaling_pathway (P06213) | 1 | 0.6 | 1 | Alzheimer disease-presenilin pathway (P00004) | 1 | 2.0 |
| 2 | DPP-SCW_signaling_pathway (P06212) | 1 | 0.6 | 2 | Opioid prodynorphin pathway (P05916) | 1 | 2.0 |
| 3 | BMP_signaling_pathway-drosophila (P06211) | 1 | 0.6 | 3 | Integrin signalling pathway (P00034) | 3 | 6.0 |
| 4 | Axon guidance mediated by netrin (P00009) | 2 | 1.1 | 4 | Opioid proenkephalin pathway (P05915) | 1 | 2.0 |
| 5 | Axon guidance mediated by Slit/Robo (P00008) | 2 | 1.1 | 5 | Enkephalin release (P05913) | 1 | 2.0 |
| 6 | Metabotropic glutamate receptor group III pathway (P00039) | 1 | 0.6 | 6 | Inflammation mediated by chemokine and cytokine signaling pathway (P00031) | 3 | 6.0 |
| 7 | Axon guidance mediated by semaphorins (P00007) | 1 | 0.6 | 7 | Angiotensin II-stimulated signaling through G proteins and beta-arrestin (P05911) | 1 | 2.0 |
| 8 | Apoptosis signaling pathway (P00006) | 1 | 0.6 | 8 | Gonadotropin releasing hormone receptor pathway (P06664) | 2 | 4.0 |
| 9 | De novo purine biosynthesis (P02738) | 1 | 0.6 | 9 | Vitamin D metabolism and pathway (P04396) | 1 | 2.0 |
| 10 | Angiogenesis (P00005) | 2 | 1.1 | 10 | Vasopressin synthesis (P04395) | 1 | 2.0 |
| 11 | Ionotropic glutamate receptor pathway (P00037) | 1 | 0.6 | 11 | Cytoskeletal regulation by Rho GTPase (P00016) | 1 | 2.0 |
| 12 | Alzheimer disease-presenilin pathway (P00004) | 4 | 2.3 | 12 | Nicotinic acetylcholine receptor signaling pathway (P00044) | 1 | 2.0 |
| 13 | Interleukin signaling pathway (P00036) | 1 | 0.6 | 13 | Cadherin signaling pathway (P00012) | 3 | 6.0 |
| 14 | Alzheimer disease-amyloid secretase pathway (P00003) | 2 | 1.1 | 14 | Blood coagulation (P00011) | 14 | 28.0 |
| 15 | Interferon-gamma signaling pathway (P00035) | 1 | 0.6 | 15 | Metabotropic glutamate receptor group II pathway (P00040) | 1 | 2.0 |
| 16 | Integrin signalling pathway (P00034) | 15 | 8.6 | 16 | CCKR signaling map (P06959) | 2 | 4.0 |
| 17 | Insulin/IGF pathway-mitogen activated protein kinase kinase/MAP kinase cascade (P00032) | 1 | 0.6 | 17 | Huntington disease (P00029) | 3 | 6.0 |
| 18 | Inflammation mediated by chemokine and cytokine signaling pathway (P00031) | 13 | 7.5 | 18 | Wnt signaling pathway (P00057) | 3 | 6.0 |
| 19 | Nicotine pharmacodynamics pathway (P06587) | 1 | 0.6 | 19 | Glycolysis (P00024) | 1 | 2.0 |
| 20 | Huntington disease (P00029) | 8 | 4.6 | 20 | Toll receptor signaling pathway (P00054) | 1 | 2.0 |
| 21 | p53 pathway (P00059) | 2 | 1.1 | 21 | FAS signaling pathway (P00020) | 1 | 2.0 |
| 22 | Wnt signaling pathway (P00057) | 8 | 4.6 | 22 | Plasminogen activating cascade (P00050) | 4 | 8.0 |
| 23 | Vitamin D metabolism and pathway (P04396) | 1 | 0.6 |  |  |  |  |
| 24 | Vasopressin synthesis (P04395) | 2 | 1.1 |  |  |  |  |
| 25 | VEGF signaling pathway (P00056) | 2 | 1.1 |  |  |  |  |
| 26 | Glycolysis (P00024) | 3 | 1.7 |  |  |  |  |
| 27 | Transcription regulation by bZIP transcription factor (P00055) | 1 | 0.6 |  |  |  |  |
| 28 | General transcription regulation (P00023) | 1 | 0.6 |  |  |  |  |
| 29 | Toll receptor signaling pathway (P00054) | 2 | 1.1 |  |  |  |  |
| 30 | Ras Pathway (P04393) | 2 | 1.1 |  |  |  |  |
| 31 | Methylmalonyl pathway (P02755) | 1 | 0.6 |  |  |  |  |
| 32 | General transcription by RNA polymerase I (P00022) | 1 | 0.6 |  |  |  |  |
| 33 | T cell activation (P00053) | 2 | 1.1 |  |  |  |  |
| 34 | FGF signaling pathway (P00021) | 3 | 1.7 |  |  |  |  |
| 35 | TGF-beta signaling pathway (P00052) | 2 | 1.1 |  |  |  |  |
| 36 | FAS signaling pathway (P00020) | 1 | 0.6 |  |  |  |  |
| 37 | Plasminogen activating cascade (P00050) | 7 | 4.0 |  |  |  |  |
| 38 | Endothelin signaling pathway (P00019) | 1 | 0.6 |  |  |  |  |
| 39 | EGF receptor signaling pathway (P00018) | 4 | 2.3 |  |  |  |  |
| 40 | p38 MAPK pathway (P05918) | 1 | 0.6 |  |  |  |  |
| 41 | Parkinson disease (P00049) | 3 | 1.7 |  |  |  |  |
| 42 | Cytoskeletal regulation by Rho GTPase (P00016) | 9 | 5.2 |  |  |  |  |
| 43 | PDGF signaling pathway (P00047) | 2 | 1.1 |  |  |  |  |
| 44 | Opioid prodynorphin pathway (P05916) | 1 | 0.6 |  |  |  |  |
| 45 | Opioid proenkephalin pathway (P05915) | 1 | 0.6 |  |  |  |  |
| 46 | Nicotinic acetylcholine receptor signaling pathway (P00044) | 3 | 1.7 |  |  |  |  |
| 47 | Cadherin signaling pathway (P00012) | 6 | 3.4 |  |  |  |  |
| 48 | Enkephalin release (P05913) | 1 | 0.6 |  |  |  |  |
| 49 | Blood coagulation (P00011) | 19 | 10.9 |  |  |  |  |
| 50 | Dopamine receptor mediated signaling pathway (P05912) | 1 | 0.6 |  |  |  |  |
| 51 | Muscarinic acetylcholine receptor 1 and 3 signaling pathway (P00042) | 1 | 0.6 |  |  |  |  |
| 52 | B cell activation (P00010) | 2 | 1.1 |  |  |  |  |
| 53 | Angiotensin II-stimulated signaling through G proteins and beta-arrestin (P05911) | 2 | 1.1 |  |  |  |  |
| 54 | Metabotropic glutamate receptor group I pathway (P00041) | 1 | 0.6 |  |  |  |  |
| 55 | Metabotropic glutamate receptor group II pathway (P00040) | 1 | 0.6 |  |  |  |  |
| 56 | CCKR signaling map (P06959) | 4 | 2.3 |  |  |  |  |
| 57 | Pyruvate metabolism (P02772) | 1 | 0.6 |  |  |  |  |
| 58 | Gonadotropin releasing hormone receptor pathway (P06664) | 8 | 4.6 |  |  |  |  |
| 59 | SCW_signaling_pathway (P06216) | 1 | 0.6 |  |  |  |  |
